# Genome-wide investigation of synthetic rescue interactions in Alzheimer’s disease implicates glial lipid and sterol metabolism

**DOI:** 10.1186/s13195-026-02009-4

**Published:** 2026-04-11

**Authors:** Jun Ki Yoo, Ju Han Kim

**Affiliations:** Seoul National University Biomedical Informatics (SNUBI), Division of Biomedical Informatics, Seoul National University College of Medicine, Seoul, 03080 Republic of Korea

**Keywords:** Alzheimer’s disease, Synthetic rescue, Genetic interaction, Whole exome sequencing, Cognitive resilience, Lipid metabolism, Sterol metabolism, Glial cell

## Abstract

**Background:**

Synthetic rescue (SR) interactions, where a disease-promoting alteration in one gene is compensated by a secondary alteration in another gene, remain largely unexplored in Alzheimer’s disease (AD). Here, we performed a genome-wide investigation of SR gene pairs that mitigate the genetic risk of AD.

**Methods:**

Using whole exome sequencing (WES) data from the Alzheimer’s Disease Sequencing Project (ADSP) participants (*n* = 9,895), we first identified AD risk genes by two complementary methods: Single-variant analysis and Gene-wise Variant Burden (GVB) analysis. We then performed a genome-wide log odds ratio comparison to identify candidate SR gene pairs and further prioritized them via Cox proportional hazards regression. Validation and single-cell analyses were performed in the Religious Orders Study and Rush Memory and Aging Project (ROSMAP) cohort.

**Results:**

Among the 37 candidate SR gene pairs, 27 pairs showing significant protective effects (HR < 1, FDR < 0.05) in Cox regression were prioritized. Notably, *NR4A1*, *SULT2A1*, *AKR1C4*, *OR52H1*, *ARMC7*, *RBAK-RBAKDN*, *DMC1* for *APOE*, and *LPP*, *ZNF510* for *TREM2* reduced the hazard of AD onset by more than half. The prioritized SR pairs were validated in ROSMAP cohort using a synthetic rescue score (SRS) that quantifies the cumulative protective effect of rescuer genes against risk gene burden. We observed that SRS was significantly associated with delayed AD onset. In addition, SRS was significantly associated with better cognitive outcomes but not with neuropathological burden, suggesting that SR pairs may confer cognitive resilience. Functional enrichment and single cell analyses highlighted lipid and sterol metabolism in oligodendrocytes and astrocytes as a plausible biological mechanism of SR interactions in AD.

**Conclusions:**

Our study extends understanding of SR interactions in AD, implicating glial lipid and sterol metabolism as a key underlying mechanism and providing novel insights for therapeutic strategies beyond targeting AD risk loci.

**Supplementary Information:**

The online version contains supplementary material available at 10.1186/s13195-026-02009-4.

## Background

Alzheimer’s disease (AD), the most common cause of dementia, is characterized by progressive cognitive impairment and memory decline [1]. The pathophysiology of AD involves the accumulation of amyloid-beta (Aβ) plaques and neurofibrillary tangles (NFTs), which consequently lead to the death of neurons [2, 3]. Large scale genome-wide association studies (GWASs) have discovered over one hundred AD-associated loci including *APOE*, *BIN1*, *TREM2*, and *ABCA7* [4]. However, previous genetic studies have mainly focused on identifying AD risk loci, whereas protective variants that reduce AD risk or delay AD onset are relatively underexplored [5, 6].

**Fig. 1:**
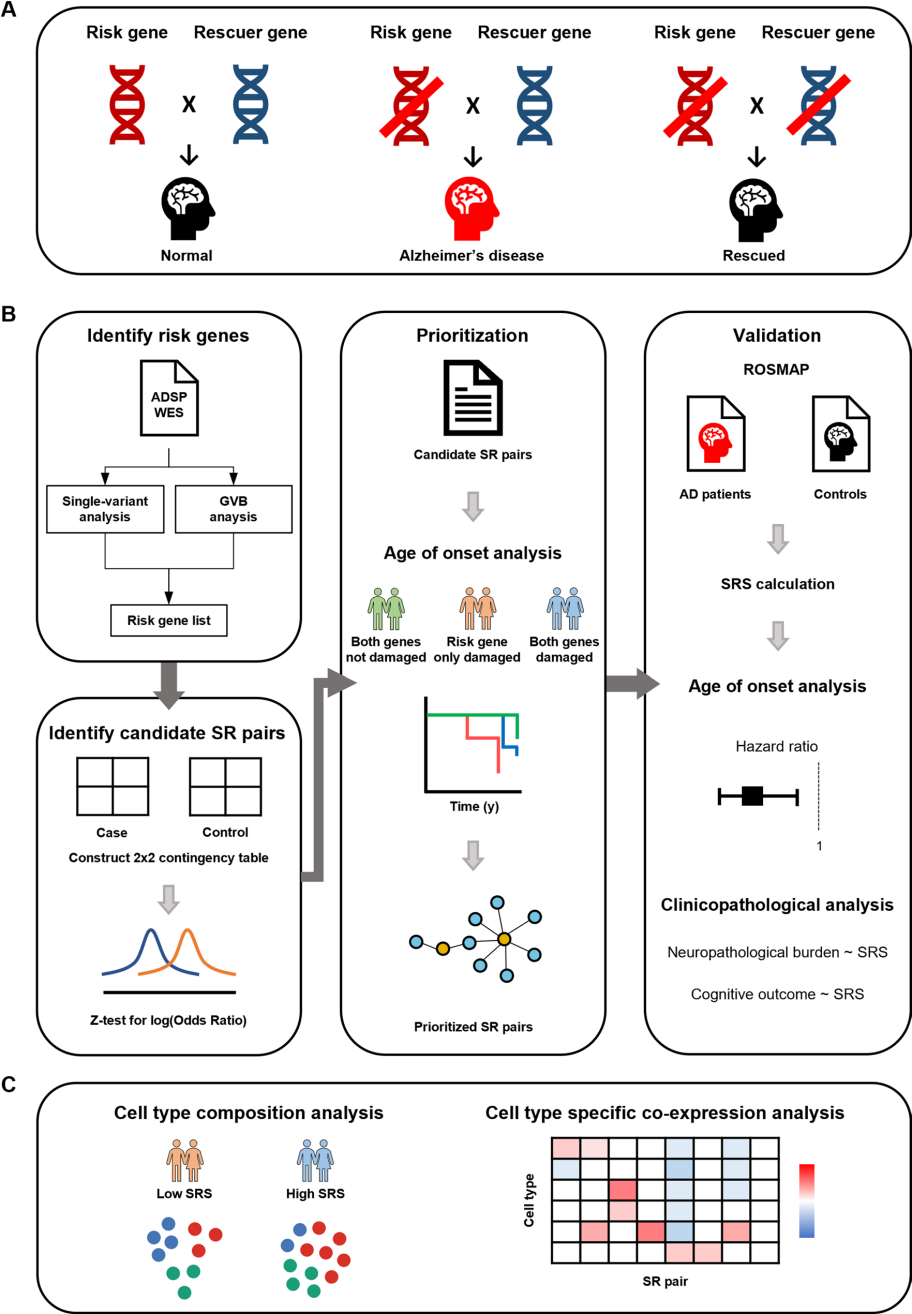
Research design and workflow of identifying SR gene pairs in AD. **A** The concept of SR gene pair. **B** Schematic overview of SR pair identification and validation. Incorporating the WES data from ADSP, we first conducted single-variant analysis and GVB analysis to identify AD risk genes. Second, we performed genome-wide screening to identify candidate rescuer genes for the AD risk genes. Third, we prioritized the SR pairs by evaluating their effects on the AD onset age. Finally, we validated the prioritized SR pairs in the ROSMAP cohort. **C** Single-cell analyses of the SR pairs

Synthetic rescue (SR), also known as genetic suppression, refers to an interaction where a disease-promoting alteration in a primary risk gene is compensated by a secondary alteration in a distinct rescuer gene [7, 8] (Fig. 1A). Genome-wide SR interactions have been systematically mapped in resistance to cancer therapy by integrating cell line and patient-level multi-omics data [9–11]. Beyond oncology, SR interactions have been widely studied in various Mendelian diseases, such as cystic fibrosis, Fanconi anemia, and Bardet-Biedl syndrome, via genome-wide RNAi or CRISPR/Cas9 screening [8, 12–14]. However, studies on SR interactions in AD remain limited. Despite the polygenic architecture of AD, most efforts to identify SR interactions in AD are confined to the *APOE* ε4 carriers [15–17]. Therefore, a genome-wide investigation of SR interactions in AD might reveal new protective mechanisms and provide evidence for novel therapeutic strategies.

In this study, we analyzed whole exome sequencing (WES) data from 9,895 participants in the Alzheimer’s Disease Sequencing Project (ADSP) to identify AD risk genes. Based on the hypothesis that concurrent damage in SR gene pairs would be more frequent in controls relative to AD cases, we then conducted a genome-wide screening to discover SR interactions between each AD risk gene and all other protein-coding genes. We further prioritized the SR gene pairs by examining their effects on AD onset using Cox regression analysis. The final set of SR pairs were validated in the Religious Orders Study and the Rush Memory and Aging Project (ROSMAP) cohort (Fig. 1B). Additionally, we integrated single-nucleus RNA sequencing (snRNA-seq) data from ROSMAP to profile SR interactions at single-cell resolution (Fig. 1C). Our study aims to uncover SR interactions in AD, characterize their cell-type specific contexts, and highlight their potential as new therapeutic targets.

## Methods

### Discovery cohort

The WES and phenotype data of the ADSP Discovery Phase were obtained from dbGaP (accession: phs000572.v8.p4). The ADSP WES and phenotype data contain total 10,913 participants in six different consent groups. We excluded 766 samples from the ROSMAP study to prevent overlap between the discovery and validation cohorts. We further removed 237 samples with discordant identifiers between genotype and phenotype data or AD status “NA”, leaving 9,910 samples (5,577 cases and 4,333 controls). The cases met the NINCDS-ADRDA criteria for possible, probable, or definite AD and the controls were cognitively normal [18].

### Data processing and quality control

We first performed variant-level quality control (QC) on a VCF file containing 1,524,414 SNVs and indels from 9,910 samples by removing variants with a missing call rate greater than 5%. We then excluded 15 samples with more than 5% missing genotype calls, resulting in 5,571 AD cases and 4,324 controls. Multi-allelic variants were split into bi-allelic records using BCFtools v1.15 [19]. Variants were annotated with hg19 reference genome databases using ANNOVAR [20]. We defined putative loss-of-function (pLOF) variants as start-loss, stop-loss, stop-gain, frameshift indels and non-frameshift indels. We retained 807,652 nonsynonymous SNVs and pLOF variants, and only variants with minor allele count (MAC) ≥ 3 were selected for subsequent analyses (Fig. S1).

Additionally, we computed principal components (PCs) using EIGENSTRAT [21, 22] in the smartpca program to adjust for population structure in association analyses. 17,905 SNVs were selected based on the following criteria: MAF ≥ 5%, call rate ≥ 99%, and only one SNV per pair of SNVs with linkage disequilibrium (LD) of r^2^ > 0.5 within a 50-SNV window.

### Identification of AD risk genes

We defined AD risk gene as the union of genes identified by single-variant analysis and Gene-wise Variant Burden (GVB) analysis. We annotated the AD risk genes with four molecular genetic features: coding sequence (CDS) length, probability of being loss-of-function intolerant (pLI), protein-protein interaction (PPI) degree, and tissue specificity index tau (τ) [23]. CDS length was obtained from BioMart [24] website (GRCh37.p13, Ensembl 115, September 2025). The pLI was extracted from the study of Monkol et al. [25]. PPI degree was calculated using physical interaction data from BioGRID database (version 4.4.222) [26]. Tau (τ) values were obtained from Palmer et al. [27]. The features of gene groups identified by two different methods were compared using Wilcoxon rank-sum test.

### Single-variant analysis

We performed single-variant analysis to identify variants associated with AD. Multivariable logistic regression was conducted under two models of inheritance (dominant and recessive), adjusting for sex, sequencing center, and the top 10 PCs. The Benjamini-Hochberg false discovery rate (FDR) correction was performed, with FDR < 0.05 considered statistically significant. Genes containing variants significantly associated with AD were defined as AD risk genes.

### Gene-wise variant burden analysis

Gene-wise Variant Burden (GVB) [28] is a scoring method to predict the cumulative genetic effect of coding variants within a gene. To calculate GVB, we included only nonsynonymous SNVs with SIFT [29] score < 0.7 and pLOF variants. The GVB score of a gene for each individual was calculated as the geometric mean of SIFT. Homozygous variants were considered more damaging and therefore the square of SIFT score was used instead. When the SIFT score of the variant was zero or the variant was pLOF, we assigned a near-zero value (10^− 8^) to avoid multiply-by-zero problem. For each gene, multivariable logistic regression was performed to assess the association between GVB scores and AD status, adjusting for the same covariates used in the single-variant analysis. Genes with cumulative minor allele count (cMAC) > 10 and at least two contributing variants were included in this analysis. Genes with FDR < 0.05 were considered statistically significant and defined as AD risk genes.

### SR pair screening using log odds ratio comparison

To identify candidate SR gene pairs for AD risk genes, each risk gene was paired with all other genes. For each gene pair, we constructed 2 by 2 contingency table in AD cases and control group respectively. Each contingency table classified individuals based on the damage status (damaged vs. not damaged) of both the risk gene and the paired rescuer gene. Individuals who did not meet the criteria for being classified as “damaged” were considered “not damaged”.

The “risk gene damaged” status was defined using two approaches: (i) For risk genes identified by the single-variant analysis, individuals carrying at least one copy of the risk allele were classified as “risk gene damaged”. Here, the risk allele was defined as the alternative allele if the odds ratio (OR) from the single-variant analysis was greater than 1; otherwise, if the OR was less than 1, the reference allele was defined as the risk allele. (ii) For risk genes identified by the GVB analysis, definition depended on the direction of the association. If the OR for the GVB score was less than 1, individuals with GVB < 0.2 were classified as “risk gene damaged”; otherwise, if the OR was greater than 1, individuals with GVB ≥ 0.2 were classified as “risk gene damaged”. For risk genes identified by both analyses, both approaches were applied independently. The 0.2 threshold was selected based on the genome-wide distribution of GVB values and its ability to discriminate the damage status of two well-known AD risk genes, *APOE* and *TREM2* (Fig. S2). The paired rescuer gene was defined as damaged in individuals with GVB < 0.2 for that gene.

We then calculated the ORs from the contingency tables of AD cases and controls for each pair, followed by log-transformation to approximate normal distribution for statistical testing. The natural logarithms of ORs between AD cases and controls were compared using one-sided Z-test, testing for higher value in controls. Significant pairs (FDR < 0.1) from either approach were considered candidate SR pairs and passed to the next analysis.

### Age of onset analysis

We further filtered and selected SR pairs that significantly affect the age of onset of AD. Cox proportional hazards regression was used to assess the effect of a rescuer gene on the AD onset. For each candidate SR pair, individuals were categorized into four groups based on the status of a risk gene and its rescuer gene: (i) Both genes not damaged, (ii) Risk gene only damaged, (iii) Rescuer gene only damaged (iv) Both genes damaged. The “Risk gene only damaged” group (ii) was set as the reference. Hazard ratios (HRs) were then calculated by comparing each group to the reference group, while adjusting for sex, sequencing center, and the top 10 PCs. We focused on the comparison between “Risk gene only damaged” group (ii) and “Both genes damaged” group (iv), considering the significant (FDR < 0.05) SR pairs with HR < 1 as the final set of SR pairs. To evaluate the additive effect of multiple rescuer genes for *APOE*, we conducted additional Cox regression analysis on the number of SR pairs adjusting for sex, sequencing center, and the top 10 PCs. This analysis was restricted to carriers of rs429358 (classified as *APOE*-damaged). All Cox regression analyses were performed using the R package survival (version 3.5-7).

### Sensitivity analysis

To assess the robustness of the final SR pairs, we repeated SR pair screening and age of onset analysis using alternative GVB thresholds of 0.1 and 0.3. SR pairs identified at the primary threshold (0.2) were evaluated for replication at each alternative threshold, and the overlap was visualized using the R package ComplexHeatmap (v2.18.0) [30].

### External validation cohort

To validate the final set of SR pairs, we obtained WGS and clinical data of two longitudinal cohort studies: the ROS and MAP [31]. The ROS and MAP are complementary studies of aging and AD conducted by Rush University. ROS enrolled individuals from religious communities (nuns, priests, and brothers) across the United States. MAP recruited participants from retirement communities in northeastern Illinois to include individuals with broader life experiences and socioeconomic backgrounds. Both studies required participants without any signs of dementia at the time of enrollment. The participants also agreed to receive annual cognitive assessments and to donate brain after death. WGS VCF files and clinical data were downloaded from the AD Knowledge Portal (Synapse ID: syn10901595 and syn3157322).

Among the 1,196 individuals with available WGS data, we excluded duplicates, discordant samples, and non-white samples. Participants were then selected based on the clinical diagnosis of cognitive status at last visit. Those diagnosed with AD or AD+ were classified as cases (*n* = 480) and individuals with no cognitive impairment (NCI) were classified as controls (*n* = 381). The WGS VCF of 861 participants then underwent split of multi-allelic variants, annotation, selection of nonsynonymous SNVs and pLOF variants, and PC computation as described in the *Data processing and quality control* section.

### Synthetic rescue score analysis

We defined the synthetic rescue score (SRS) to quantify the cumulative effect of the final set of SR pairs in the ROSMAP cohort. We first computed rescued-hazard score (RHS) for each risk gene in each individual by leveraging HRs obtained from the age of onset analysis in the ADSP discovery cohort, normalizing by the number of rescuer genes to prevent overestimation of risk gene effect.

For the j-th risk gene in the i-th individual, RHS_*ij*_ was defined as:

$$RH{S}_{ij}\left\{\begin{array}{ll}\mathrm{0}, & if\;g_{j}=0\\ \frac{1}{k}\sum_{x=1}^{k}\left[\log\left({HR}_{j}\right)+\delta\left(x\right)\cdot\log\left({HR}_{j,x}\right)\right], & if\;g_{j}=1\end{array}\right.$$

where *g*_*j*_ = 1 if the j-th risk gene is damaged and 0 otherwise, *k* denotes the number of rescuer genes for the risk gene, *δ*(*x*) indicates whether the *x*-th rescuer gene is damaged, HR_*j*_ is the HR of “Risk gene only damaged” group compared to the “Both genes not damaged” group, and HR_*j*,*x*_ is the HR of “Both genes damaged” group compared to the “Risk gene only damaged” group.

The individual-level score RHS_*i*_ was obtained by summing the RHS_*ij*_ values:

$$RH{S}_{i}=\sum\limits_{j=1}^{N}RH{S}_{ij}$$

SRS was then computed for individuals with RHS_*i*_ > 0, indicating the presence of at least one damaged risk gene, to avoid potential confounding effects unrelated to synthetic rescue. We derived SRS by z-normalizing the RHS_*i*_ values and reversing the sign, such that a higher SRS reflects a greater synthetic rescue effect despite risk gene damage:

$$SRS=-\left(\frac{{RHS}_{i}-\mu}{\sigma}\right)({RHS}_{i}>0)$$

We evaluated the clinical relevance of SRS by assessing its association with AD onset, neuropathological burden, and cognitive outcomes. For the age of AD onset, we performed Cox regression adjusting for sex, years of education, and the top four PCs. We further examined the association of SRS with Braak stages [32] and CERAD scores [33]. Braak stages were grouped into four categories: 0, I-II, III-IV, and V-VI. We performed multivariable ordinal logistic regression for both Braak groups and CERAD scores, adjusting for age at death, sex, years of education, and the top four PCs. We also assessed the association of SRS with Mini-Mental State Examination (MMSE) scores at last visit and final consensus cognitive diagnosis while accounting for neuropathological burden. For MMSE scores, we conducted multivariable linear regression adjusting for age at last visit, sex, years of education, Braak groups, CERAD scores and the top four PCs. For final consensus cognitive diagnosis, we classified individuals diagnosed with AD or AD + as cases (*n* = 169) and individuals with NCI as controls (*n* = 61). We performed multivariable logistic regression adjusting for age at death, sex, years of education, Braak groups, CERAD scores and the top four PCs.

### Functional and phenotype enrichment analysis

We explored the functional and phenotype enrichment of AD risk genes and rescuer genes using the R package enrichR (version 3.2) [34]. GO_Biological_Process_2023, GO_Molecular_Function_2023, GO_Cellular_Component_2023, GWAS_Catalog_2023, KEGG_2021_Human, and Reactome_2022 databases were selected for the analyses. We focused on the top five enriched terms with p-value < 0.05 from each database.

### snRNA-seq analyses

Post-mortem frozen dorsolateral prefrontal cortex (DLPFC, BA9) specimens were obtained from 465 ROSMAP participants with RNA integrity number (RIN) > 5 and post-mortem interval (PMI) < 24 h. Participants without WGS data or with a low number of nuclei were excluded, resulting in 437 participants with at least 868 nuclei assigned [35]. The final data consisted of seven major cell types, including astrocytes, excitatory neurons, inhibitory neurons, microglia, oligodendrocytes, oligodendrocyte precursor cells (OPCs), and vascular niche. snRNA-seq data were obtained from the AD Knowledge Portal (Synapse ID: syn31512863). We applied additional filters by removing cells with less than 200 or more than 20,000 total RNA Unique Molecular Identifier (UMI) counts, or having more than 5% mitochondrial mapped reads.

Among the 437 participants with snRNA-seq data, we retained 86 individuals with SRS values for cell type composition analysis. One participant without PMI data was then removed. The association of SRS with cell type composition was assessed using the propeller method from the speckle R package (v1.2.0) [36] with limma (v3.58.1) [37]. Analyses were performed at two levels of cellular resolution: three major cell classes (neurons, glia and vascular niche) and seven major cell types (excitatory neurons, inhibitory neurons, astrocytes, microglia, oligodendrocytes, OPCs, and vascular niche). In both analyses, cell type proportions were logit-transformed and modeled using a linear model framework, adjusting for age at death, sex and PMI. Cell types with FDR < 0.05 were considered statistically significant.

Cell-type specific co-expression of SR pairs was analyzed using the R package CS-CORE (v1.0.2) [38] across 437 participants. Within each major cell type, pairwise correlation coefficients were calculated for genes in SR pairs using raw UMI counts. The Benjamini-Hochberg FDR correction was applied and gene pairs with FDR < 0.05 were considered statistically significant. The *AKR1C4* and *RBAK-RBAKDN* genes were excluded from the analysis due to unavailable UMI counts in all major cell types.

## Results

### Single-variant and GVB analyses identify AD risk genes

As SR denotes an interaction between a disease risk gene and a rescuer gene, we first performed single-variant and GVB analyses on 5,571 AD cases and 4,324 controls to identify risk genes associated with AD. A total of 174,588 nonsynonymous SNVs and pLOF variants with MAC ≥ 3 were included in the analyses (Fig. S1).

In the single-variant analysis, 19 nonsynonymous SNVs across 11 genes were significant at FDR < 0.05, including one variant in *TREM2* (rs75932628), one in *TNFRSF10A* (rs2230229), one in *MS4A6A* (rs7232), one in *WNK1* (rs7955371), four in *SPPL2C* (rs12185233, rs12185268, rs12373123, rs12373139), three in *MAPT* (rs62063786, rs62063787, rs10445337), two in *KANSL1* (rs34579536, rs34043286), one in *OR1I1* (rs8104843), one in *CBLC* (rs3208856), two in *BCAM* (rs28399653, rs28399654), and two in *APOE* (rs440446, rs429358) (Table S1). Among these, rs75932628 in *TREM2* and rs429358 in *APOE* (the *APOE* ε4-defining variant) are well-established AD risk variants [39], both of which showed strong associations (OR = 4.73, *p* = 4.75e-09; OR = 4.40, *p* = 1.47e-166) under dominant model.

**Fig. 2:**
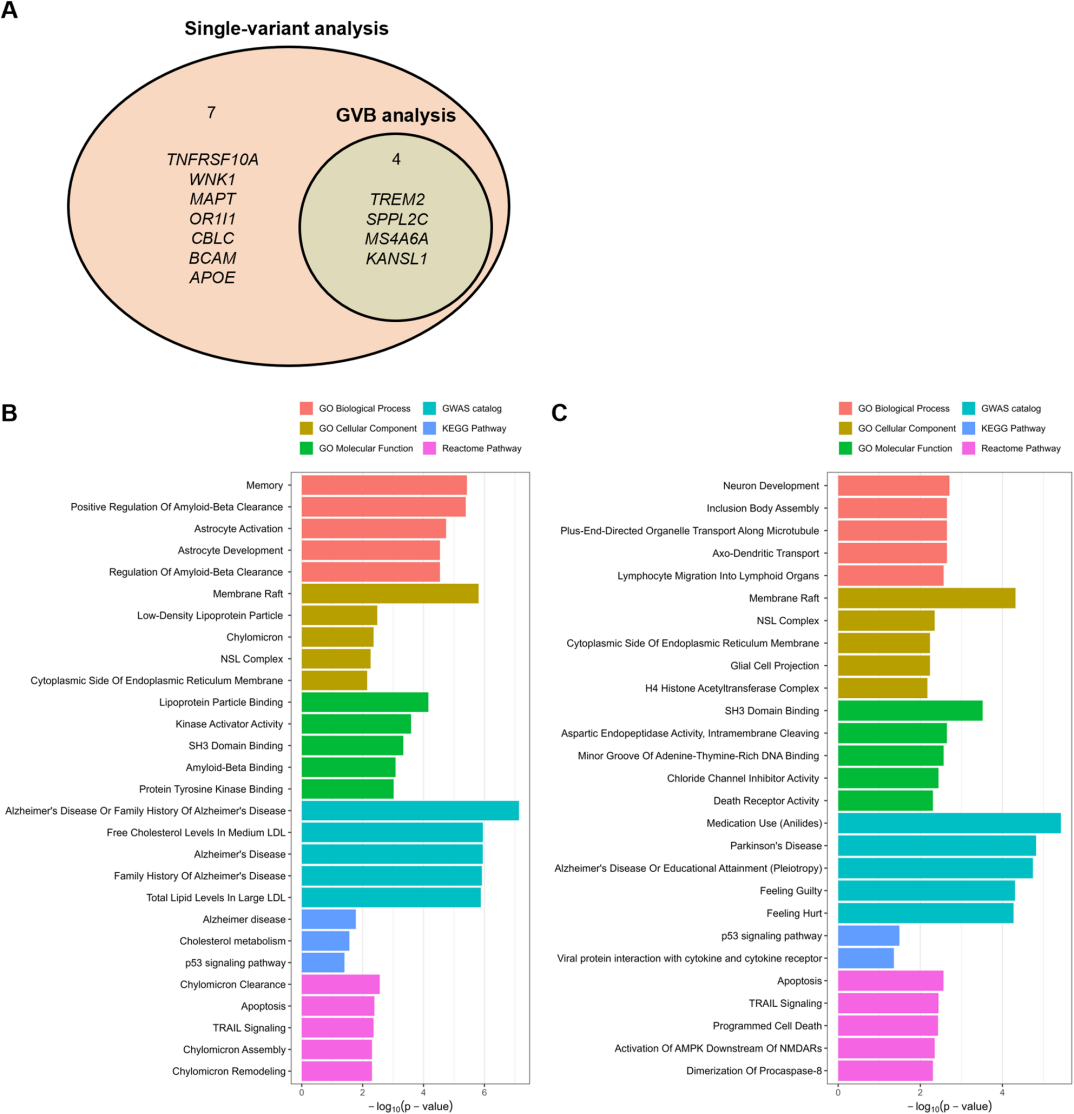
AD risk genes identified in the ADSP cohort. **A** Venn diagram shows the AD risk genes identified by single-variant analysis and GVB analysis. **B-C** Functional enrichment analysis results of the AD risk genes including all AD risk genes (**B**) and excluding *APOE* and *TREM2* (**C**). GO, Gene Ontology; GWAS, Genome-Wide Association Study; KEGG, Kyoto Encyclopedia of Genes and Genomes

GVB analysis identified four genes significantly associated with AD at FDR < 0.05, including one gene (*TREM2*) with lower mean GVB in AD cases and three genes (*MS4A6A*, *SPPL2C*, *KANSL1*) with lower mean GVB in controls (Fig. S3). These four genes were also identified in single-variant analysis (Fig. 2A), indicating that they contribute to AD risk through a single significant variant as well as cumulative variant burden effects.

To explore the molecular characteristics of AD risk genes identified by the two analyses, we categorized AD risk genes into genes identified by single-variant analysis alone (SV-only, *n* = 7) and genes identified by both analyses (Both, *n* = 4). We then compared four molecular genetic features that are associated with gene essentiality: CDS length, pLI, PPI degree, and tau (τ). Genes with longer CDS length tend to be more evolutionarily conserved and are more likely to be essential [40, 41]. pLI is a gene-level metric which represents the probability of being loss-of-function (LOF) intolerant [25], where higher values reflect stronger selective constraint against LOF variants - a hallmark of essential genes. Essential genes also tend to be more central in biological networks and therefore exhibit higher PPI degree [42]. Tau (τ) quantifies tissue specificity of gene expression, ranging from 0 (widely expressed) to 1 (highly tissue-specific). Essential genes tend to be broadly expressed across tissues and therefore exhibit lower tau values [27]. Genes in the SV-only group tend to have longer CDS length (*p* = 0.4), higher PPI degree and median pLI (*p* = 0.16; *p* = 0.4), whereas tau were relatively lower (*p* = 0.59) compared to the Both group (Fig. S4). These results suggest that SV-only genes are more likely to be essential, implying the complementary roles of the two methods in capturing the genetic architecture of AD.

We then performed functional and phenotype enrichment analysis to investigate the biological context of AD risk genes. AD risk genes were enriched in the biological processes of regulation of Aβ clearance and astrocyte activation; cellular components of membrane raft and low-density lipoprotein particle; molecular functions of lipoprotein particle binding and amyloid-beta binding; and the GWAS phenotype of AD or family history of AD and free cholesterol levels in medium LDL (Fig. 2B). Given that *APOE* and *TREM2* are well-established AD risk genes, we repeated the enrichment analysis after excluding these two genes (Fig. 2C). Membrane raft and AD-associated phenotype remained significant. Notably, neuronal terms including neuron development and axo-dendritic transport newly emerged, suggesting that AD risk genes identified in the ADSP cohort are implicated in diverse aspects of AD pathogenesis.

### Log odds ratio comparison uncovers candidate SR pairs

**Fig. 3:**
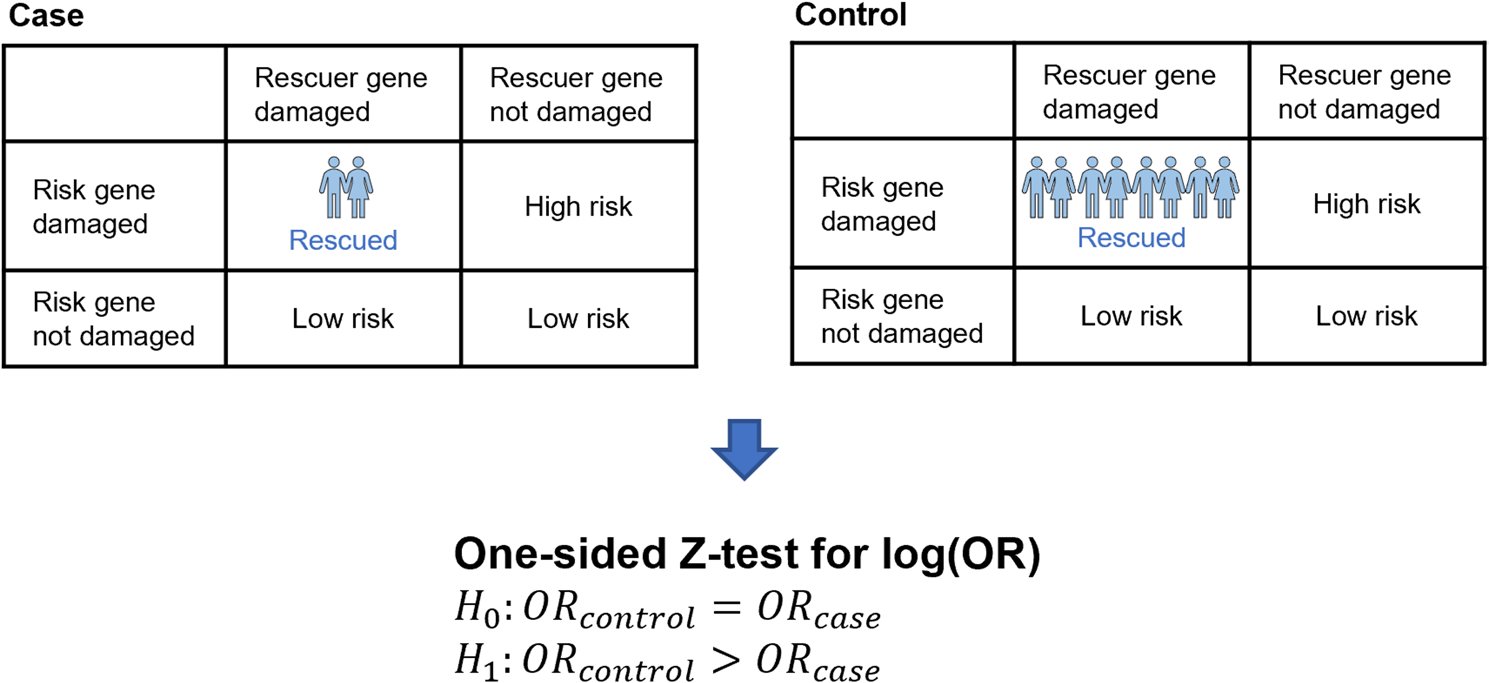
A schematic diagram of identifying candidate SR pairs. The approach compares odds ratios between AD cases and controls for each risk gene-rescuer gene combination. When a gene functions as a rescuer for a risk gene, “Both gene damaged” individuals would be enriched in controls compared to cases. One-sided Z-test is used to assess the statistical significance of the rescue effect

We next investigated candidate rescuer genes for each AD risk gene. Initially, each AD risk gene was paired with all other protein-coding genes. For each gene pair, 2 × 2 contingency tables were constructed in AD cases and control separately based on individuals’ damage status of the risk gene and its paired gene (Fig. 3). To test whether “Both genes damaged” individuals are enriched in controls compared to AD cases, we performed one-sided Z-test for log-transformed odds ratio of case and control.

The comparison yielded 37 candidate SR pairs at FDR < 0.1 (Table S2). Of these, 35 pairs included *APOE* as the risk gene and two pairs included *TREM2*, sharing one rescuer gene *LPP*. Specifically, all 35 *APOE* pairs were based on the rs429358 variant identified by single-variant analysis and two *TREM2* pairs were based on the GVB-based damage definition.

### Age of onset analysis prioritizes SR pairs

**Fig. 4:**
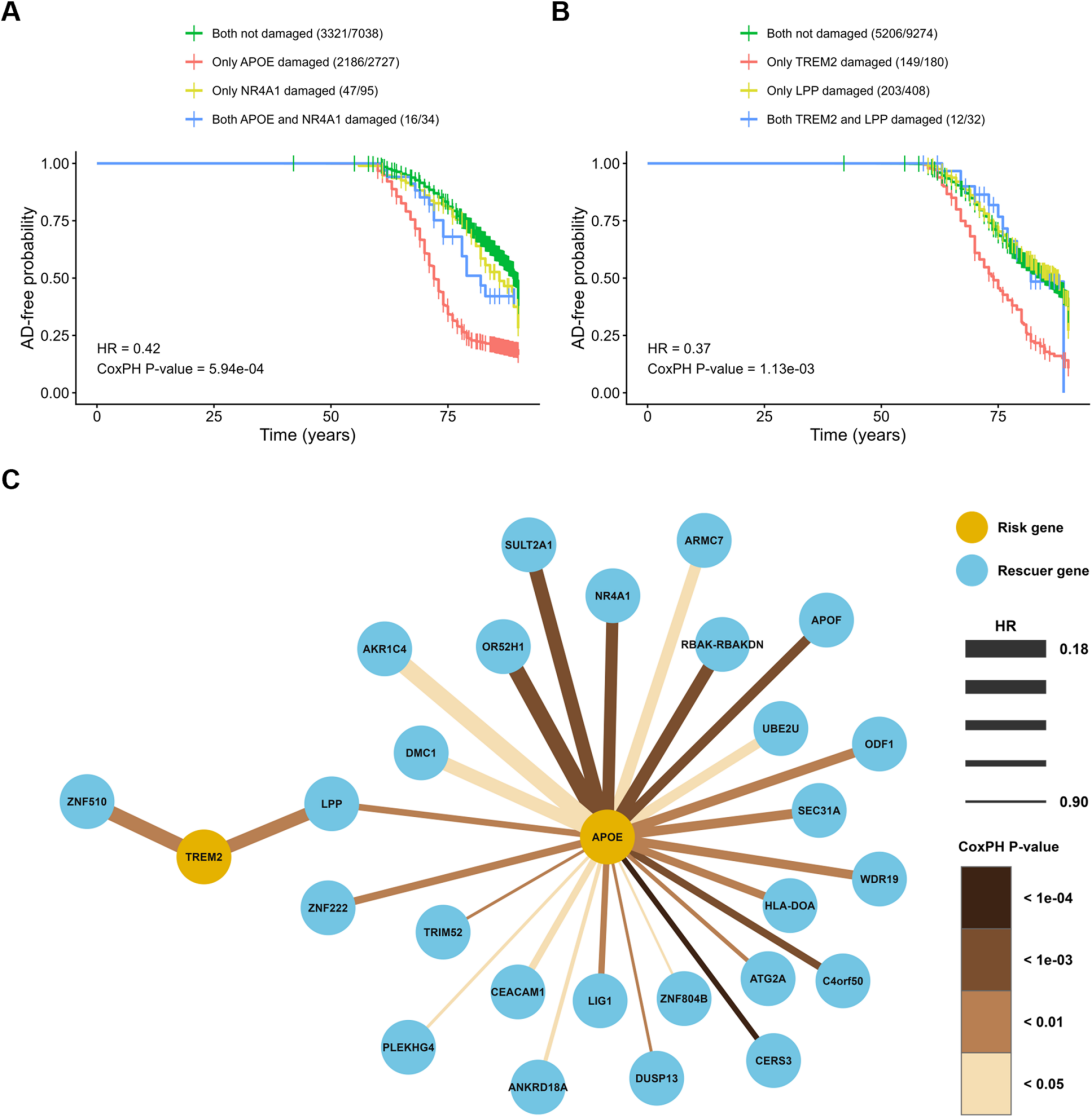
Prioritization of SR pairs. **A-B** Examples of age of onset analysis-significant (FDR < 0.05) SR pairs for *APOE* (**A**) and *TREM2* (**B**). Cox proportional hazards regression was used to obtain the HRs and the *p*-values. Each group is annotated with (The number of AD patients in the group / The total number of individuals in the group). **C** 27 prioritized SR pairs with rescuer genes (blue) and their target risk genes (yellow). Edge width and edge color represent HR and p-value obtained from Cox proportional hazards regression, respectively. HR, hazard ratio; CoxPH, Cox proportional hazards regression

We further prioritized the candidate SR pairs by examining their impact on the onset age of AD. For each candidate SR pair, we performed Cox proportional hazards regression adjusting for sex to assess whether individuals in “Both genes damaged” group exhibit significantly lower hazards than “Risk gene only damaged” group. Among the 37 candidate SR pairs, 27 (73%) showed significant protective effects (HR < 1, FDR < 0.05) on AD onset. These included 25 rescuer genes (*OR52H1*, *CERS3*, *RBAK-RBAKDN*, *APOF*, *ODF1*, *ZNF222*, *NR4A1*, *PLEKHG4*, *SULT2A1*, *LPP*, *WDR19*, *CEACAM1*, *TRIM52*, *ARMC7*, *C4orf50*, *LIG1*, *ANKRD18A*, *AKR1C4*, *UBE2U*, *SEC31A*, *DMC1*, *DUSP13*, *ZNF804B*, *ATG2A*, *HLA-DOA*) for *APOE* and two (*ZNF510*, *LPP*) for *TREM2*, with *LPP* serving as a shared rescuer gene (Fig. 4 and Fig. S5). Notably, nine rescuer genes including *OR52H1* (HR = 0.22, *p* = 2.45e-04), *RBAK-RBAKDN* (HR = 0.40, *p* = 9.33e-04), *NR4A1* (HR = 0.42, *p* = 2.20e-02), *SULT2A1* (HR = 0.34, *p* = 8.94e-04), *ARMC7* (HR = 0.45, *p* = 3.56e-02), *AKR1C4* (HR = 0.18, *p* = 1.45e-02), *DMC1* (HR = 0.34, *p* = 1.85e-02) for *APOE*, and *ZNF510* (HR = 0.28, *p* = 2.73e-03), *LPP* (HR = 0.37, *p* = 1.13e-03) for *TREM2* reduced the hazard of AD onset by more than half, indicating their strong protective effects for the risk pairs.

As *APOE* harbors multiple rescuers, we also evaluated their additive effect. The number of damaged rescuer genes for *APOE* was significantly associated with a lower hazard of AD onset (HR = 0.88, *p* < 0.001; Fig. S6).

### SR pairs show consistent protective effects across GVB thresholds

Having prioritized 27 SR pairs with significant protective effects on AD onset, we evaluated the consistency of these associations across alternative GVB thresholds of 0.1 and 0.3. Of the 27 pairs, 23 (85.2%) retained significance under at least one alternative threshold, with 16 (59.3%) remaining significant across all three thresholds (Fig. S7). Among the nine SR pairs with strong protective effects (HR < 0.5), eight (*APOE*-*OR52H1*, *APOE*-*RBAK-RBAKDN*, *APOE*-*NR4A1*, *APOE*-*SULT2A1*, *APOE*-*ARMC7*, *APOE*-*AKR1C4*, *APOE*-*DMC1*, and *TREM2*-*LPP*) replicated at either alternative threshold, with four (*APOE*-*OR52H1*, *APOE*-*RBAK-RBAKDN*, *APOE*-*NR4A1*, and *APOE*-*SULT2A1*) across all thresholds.

### External validation of SR pairs

**Fig. 5:**
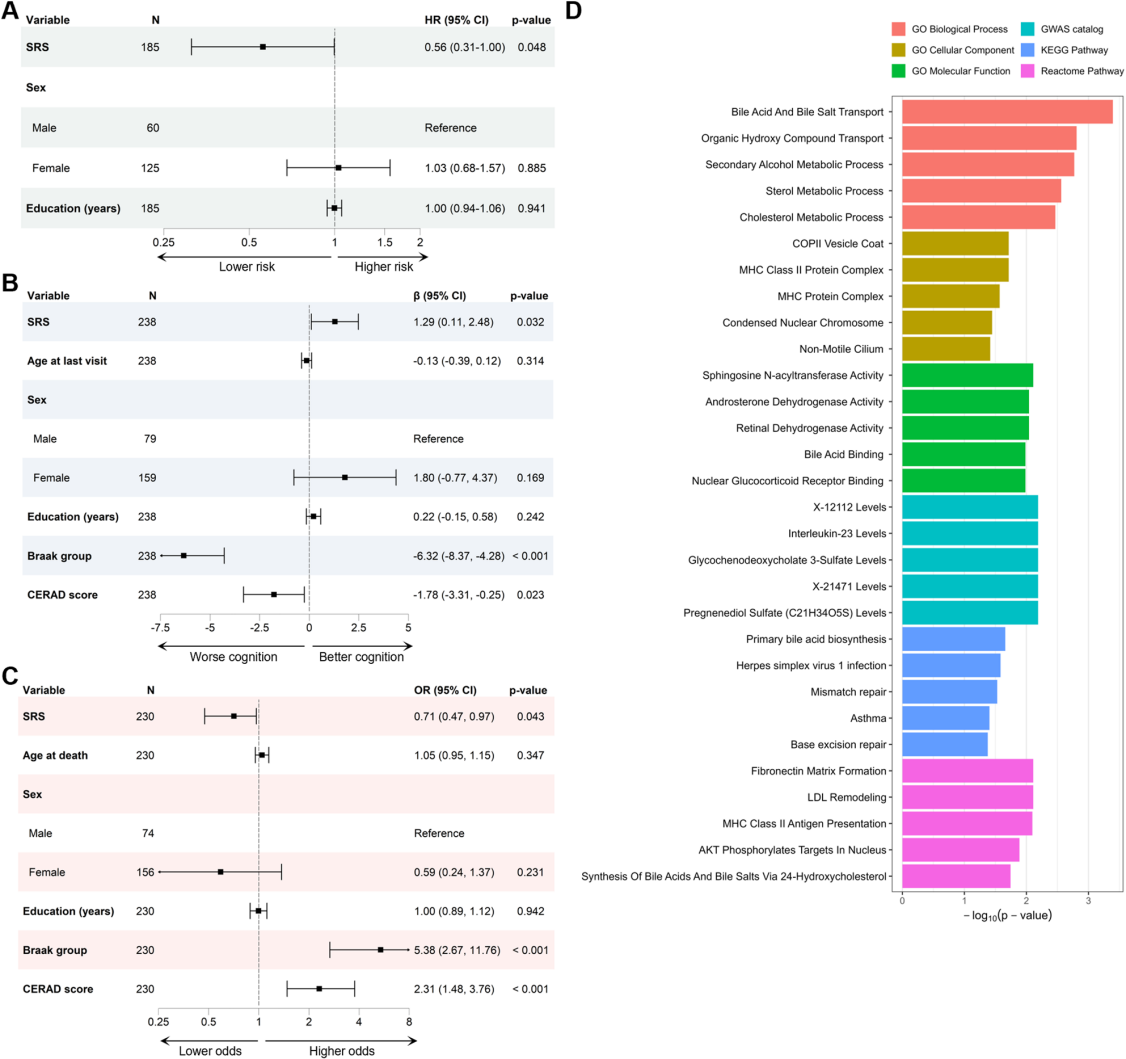
Validation of SR pairs in ROSMAP cohort. **A** Association between SRS and AD onset. Cox proportional hazards regression was used to obtain the HR and the p-value. **B**-**C** Association between SRS and MMSE score at last visit (**B**) and between SRS and final consensus cognitive diagnosis (**C**), while accounting for Braak groups and CERAD score. **D** Functional enrichment result of the rescuer genes

To validate the final set of SR pairs, we analyzed whole genome sequencing (WGS) data from 480 AD cases and 381 controls in the ROSMAP cohort. We first computed a synthetic rescue score (SRS) that quantifies the cumulative protective effect of SR pairs while accounting for risk gene effects, based on individual’s SR pair status and the corresponding HRs from the age of onset analysis (see Methods). Here, a higher SRS indicates a greater cumulative rescue effect despite risk gene damage. As expected, Cox proportional hazards regression analysis revealed that the SRS was significantly associated with a reduced risk of AD onset (HR = 0.56, *p* = 0.048; Fig. 5A).

We further investigated the relevance of SRS with AD neuropathology and cognitive outcomes. SRS did not show significant association with Braak groups (*p* = 0.76) and CERAD scores (*p* = 0.92). However, we found that SRS was significantly associated with higher MMSE score at last visit (β = 1.29; *p* = 0.032; Fig. 5B) and reduced odds of clinical AD diagnosis (OR = 0.71; *p* = 0.043; Fig. 5C) even after accounting for neuropathological burden. These results suggest that SR pairs protect cognition through alternative pathways rather than by reducing neuropathological burden.

We then explored the biological context of rescuer genes. Rescuer genes were primarily enriched in lipid- and sterol-related terms: biological processes of bile acid and bile salt transport and sterol metabolic process; molecular functions of sphingosine N-acyltransferase activity and bile acid binding; KEGG pathway of primary bile acid biosynthesis and Reactome pathway of LDL remodeling (Fig. 5D). Since AD risk genes in SR pairs (*APOE* and *TREM2*) are closely associated with lipid and sterol metabolism [39, 43], the enrichment of rescuer genes in these terms is biologically plausible and suggests that SR pairs function within shared metabolic processes.

### Single-cell analyses reveal the cellular contexts of SR pairs

**Fig. 6:**
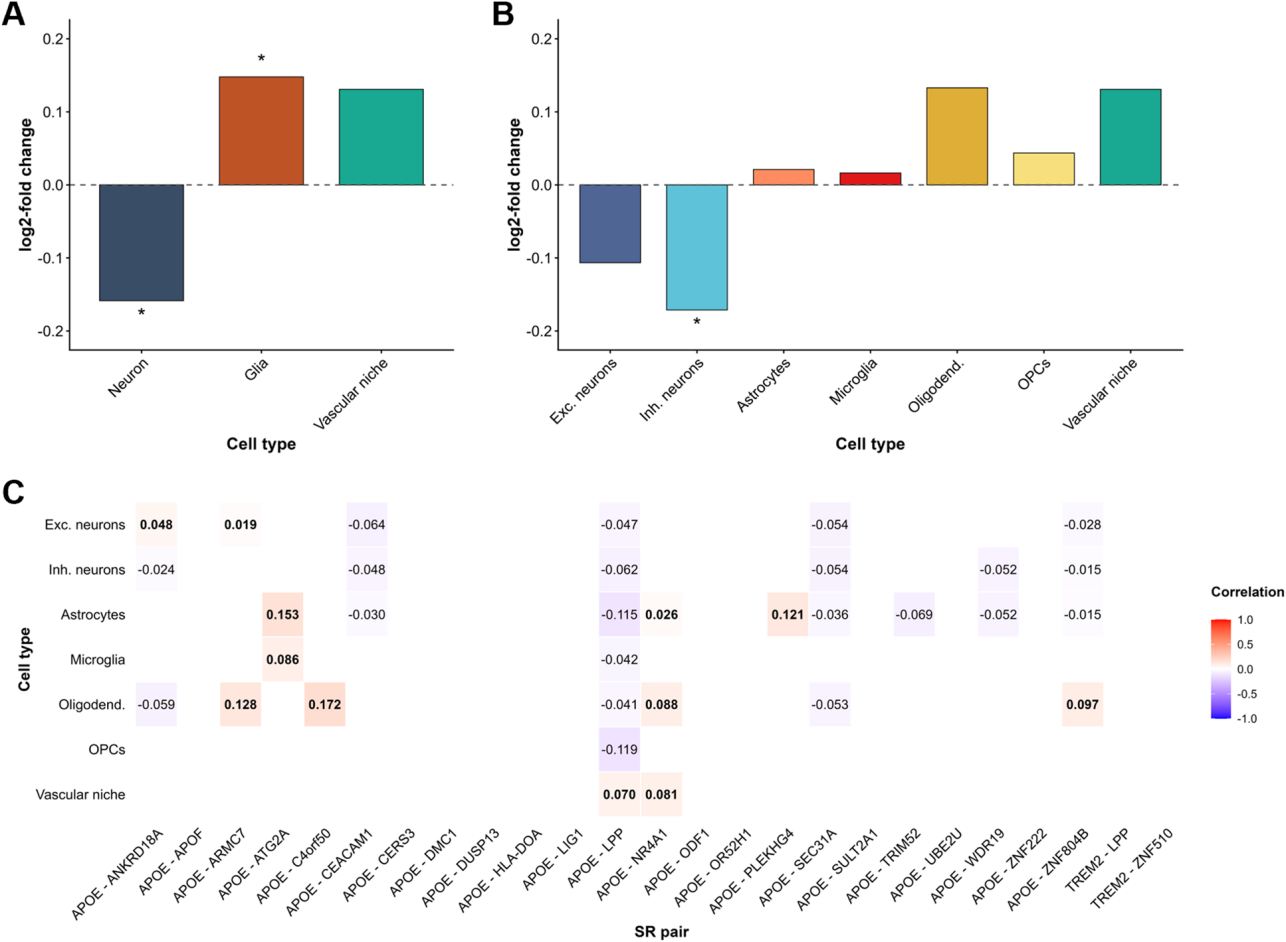
Single-cell analyses of SR pairs in the ROSMAP cohort. **A-B** Compositional shifts in brain cell types associated with SRS. Bar plots show the log2 fold change (log2FC) in cell type abundance at the major cell class level (**A**) and at the major cell type level (**B**). Significant (FDR < 0.05) results are annotated with asterisks. **C** Heatmap depicting co-expression coefficients of SR pairs in eight major cell types. Only significant (FDR < 0.05) results are shown. Positive correlations are highlighted in bold. Exc. neurons, excitatory neurons; Inh. neurons, inhibitory neurons; Oligodend., oligodendrocytes; OPCs, oligodendrocyte precursor cells

We then performed single cell analyses to characterize the cellular contexts in which the SR pairs operate. Cell type composition analysis was performed to examine the association between SRS and the abundance of brain cell types. At the major cell class level, SRS was significantly associated with an increased proportion of glia (log2FC = 0.15; FDR = 0.023) and a decreased proportion of neurons (log2FC = -0.16; FDR = 0.023) (Fig. 6A). At the major cell type level, SRS was significantly associated with a decreased proportion of inhibitory neurons (log2FC = -0.17; FDR = 0.003) (Fig. 6B). Although not significant, all four glial cell types (astrocytes, microglia, oligodendrocytes and OPCs) showed consistent positive association with SRS. These results suggest that SR pair-mediated protection mechanisms may involve glial cell populations.

Since genetic interaction pairs (e.g. synthetic lethal, synthetic rescue) are likely to be co-expressed [8, 44, 45], we further examined the cell-type specific co-expression of genes in each SR pair. Both positive and negative correlations were observed across all seven major cell types, with five cell types harboring at least one SR pair with significant positive co-expression (Fig. 6C). Among the cell types, oligodendrocytes showed the highest number of SR pairs with significant positive co-expression (4 pairs), followed by astrocytes (3 pairs). Of note, two *APOE*-containing SR pairs with strong protective effects on AD onset (HR < 0.5) showed significant positive co-expression in oligodendrocytes: *APOE*-*ARMC7* (ρ = 0.128, FDR = 8.14e-04) and *APOE*-*NR4A1* (ρ = 0.088, FDR = 3.57e-06). The positive co-expression of *APOE*-*NR4A1* was also observed in astrocytes (ρ = 0.026, FDR = 1.64e-09) and vascular niche (ρ = 0.081, FDR = 3.10e-07). Together, our analyses demonstrate that oligodendrocytes and astrocytes are the primary cellular contexts in which SR pairs exert their protective effects.

## Discussion

In this study, we investigated genome-wide SR interactions in AD by analyzing genomic and single-cell transcriptomic data. We employed odds ratio comparison to identify candidate SR pairs under the hypothesis that co-occurrence of damage in SR gene pairs would be enriched in controls compared to AD cases. We further prioritized candidate SR pairs by assessing their effects on delayed onset of AD, resulting in 27 SR pairs including the canonical AD risk genes *APOE* and *TREM2*. These findings provide novel insights into genetic interactions that mitigate the deleterious effect of AD genetic risk factors.

To establish a comprehensive pool of AD risk genes for SR mapping, we adopted two complementary approaches: single-variant analysis and GVB analysis. While single-variant analysis detects individual variant effects, GVB analysis captures the cumulative effect of all functional variants in a gene. We yielded total 11 AD risk genes, including repeatedly reported risk genes such as *APOE*, *TREM2*, *MS4A6A* and *CLU* [4, 39, 46]. Molecular genetic features and AD-related functional enrichment terms support both comprehensiveness and validity of the combined approach.

Nine rescuer genes showed considerable protective effects (HR < 0.5) for AD risk genes: *NR4A1*, *SULT2A1*, *AKR1C4*, *OR52H1*, *ARMC7*, *RBAK-RBAKDN*, *DMC1* for *APOE*, and *LPP*, *ZNF510* for *TREM2*. *NR4A1*, which encodes a member of the NR4A orphan nuclear receptor family, is involved in various biological processes including glucose and lipid metabolism, inflammatory response, cell proliferation, and apoptosis [47, 48]. Previous research has reported that *NR4A1* plays a crucial role in mitigating lipid-driven inflammation, as its deficiency in ApoE^-/-^ backgrounds substantially exacerbates lipid accumulation and pro-inflammatory signaling in atherosclerosis models [49]. Furthermore, a recent study demonstrated that targeted overexpression of *NR4A1* in aged mice ameliorates cognitive decline and enhances synaptic plasticity [50]. Since apolipoprotein E4 (apoE4) impairs glial lipid homeostasis and consequently aggravates neuroinflammation [51, 52], alterations in *NR4A1* may mitigate this apoE4-mediated lipid dysregulation and neurotoxicity. Interestingly, NR4A1 is a pharmacologically targetable nuclear receptor, and its agonist Cytosporone B (CsnB) has shown anti-inflammatory and neuroprotective effects in models of Parkinson's disease [53] and multiple sclerosis [54]. Therefore, this NR4A1-targeting compound merits further exploration for AD treatment. *SULT2A1* encodes a member of sulfotransferase family and plays an important role in steroid homeostasis [55]. Previous study found that the ratios of steroid sulfates to steroids were decreased in AD patients probably due to reduced SULT2A1 activity [56]. In addition, the neurosteroid allopregnanolone levels were significantly lower in *APOE* ε4 allele carrying AD patients compared to non-carriers [57]. Importantly, allopregnanolone has been reported to mitigate amyloid toxicity and neuroinflammation [58]. These observations support our finding that alterations in SULT2A1 may rescue *APOE* ε4-mediated compromised neurosteroid regulation and homeostasis. Similarly, *AKR1C4*, which encodes an aldo-keto reductase, is involved in the steroidogenic processes. AKR1C enzymes are essential for the biosynthesis of neuroactive steroids including allopregnanolone [59]. *OR52H1* is an olfactory receptor gene; olfactory receptors are ectopically expressed in brain glial cells and neurons, suggesting a potential role in neurodegenerative disease pathology [60]. *RBAK-RBAKDN* encodes a transcriptional repressor [61], *DMC1* is involved in DNA repair [62], and *ARMC7* has been proposed as a novel AD biomarker [63], though their protective mechanisms against *APOE* ε4 allele require further investigation.

Among the rescuer genes for *TREM2*, *LPP* encodes a LIM domain protein which regulates actin cytoskeleton dynamics and cell migration [64]. *TREM2* is predominantly expressed in microglia and is essential for amyloid plaque compaction and clearance through phagocytosis, which requires coordinated cytoskeletal remodeling [65]. Thus, *LPP* may rescue TREM2 dysfunction by facilitating phagocytic processes in microglia. *ZNF510* encodes a transcription factor, but its specific targets and mechanisms are poorly understood.

To validate the protective effect of SR pairs, we computed SRS for each individual, where higher scores indicate greater cumulative protection by rescuer genes against risk gene burden. We found that SRS is associated with a lower risk of AD onset. Notably, we observed that SRS was not significantly associated with neuropathological burden but was significantly associated with better cognitive outcomes. This finding aligns with the concept of *cognitive resilience*, where individuals maintain cognitive function despite substantial AD neuropathology [66]. Recently, van der Vliet et al. [67] reported that resilient donors exhibited lower levels of triacylglycerols and lipid droplets compared to AD, supporting the association between lipid metabolism and cognitive resilience. The enrichment of rescuer genes in lipid and sterol metabolic pathways suggests that their protective effects may be mediated by restoring the dysregulated lipid and sterol homeostasis during AD progression.

Glial cells play important roles in AD pathophysiology, contributing to Aβ clearance and degradation, suppressing neuroinflammation, and maintaining myelin integrity [68]. We observed that SRS was significantly associated with increased glial cell population, with all four glial cell types exhibiting a consistent positive trend. In cell-type specific co-expression analysis, *NR4A1* and *ARMC7* showed positive co-expression with *APOE* in oligodendrocytes. We note that oligodendrocytes play important roles in producing and maintaining myelin sheath, which contains high levels of lipids including cholesterol, phospholipids and glycolipids [69]. Furthermore, a recent study reported that *APOE* ε4 impairs myelination via cholesterol dysregulation in oligodendrocytes [70]. *NR4A1* also showed positive co-expression with *APOE* in astrocytes. Astrocytes synthesize and provide cholesterol to neurons and other glial cells via apoE to support neuronal function and myelination [71]. It has been shown that *APOE* ε4-carrying astrocytes supply excessive cholesterol to neurons, facilitating APP accumulation and Aβ formation [72]. Intriguingly, NR4A1 modulates fat and cholesterol metabolism in liver, muscle, and adipose tissue by regulating the expression of multiple lipogenic genes [73]. Together, our findings suggest that SR interactions may counteract *APOE* ε4-mediated lipid and cholesterol dysregulation within glial cells, particularly oligodendrocytes and astrocytes.

In summary, we conducted a genome-wide search for SR interactions that attenuate the effects of AD risk genes using WES/WGS data. We showed that the interactions were associated with a lower risk of AD onset as well as a later onset age. The protective effect was associated with better cognitive outcomes rather than reduced neuropathological burden, suggesting that SR pairs may confer cognitive resilience. Functional enrichment and single-cell analyses implicate restoration of lipid and sterol metabolism in oligodendrocytes and astrocytes as a plausible rescue mechanism. Our study moves beyond risk loci discovery by identifying modifier genes that mitigate the genetic susceptibility, expanding the landscape of genetic interactions and providing new insights into potential therapeutic targets in AD.

### Limitations

Our study has several limitations. First, we classified individuals based on the presence or absence of the risk allele for the genes identified by single-variant analysis, combining heterozygous and homozygous carriers into a single “risk gene damaged” status. While this approach increases statistical power, it may obscure allelic dosage effects. Second, both ADSP and ROSMAP cohorts are enriched for non-Hispanic White individuals, which may limit the generalizability of our findings to other populations. Finally, it is important to note that “rescuer gene damaged” does not necessarily imply loss-of-function of the rescuer gene. Instead, the protective effects might also result from gain-of-function. Thus, future experimental studies are needed to elucidate the precise biological mechanisms underlying each rescuer gene’s effect.

## Conclusions

In this study, we conducted a genome-wide investigation of SR gene pairs that mitigate the effect of disease-promoting alteration in AD risk genes. We detected 27 SR pairs by performing log odds ratio comparison to identify control-enriched co-occurrence of damaged gene pairs, with subsequent prioritization via age of onset analysis. We highlighted nine SR pairs, *NR4A1*, *SULT2A1*, *AKR1C4*, *OR52H1*, *ARMC7*, *RBAK-RBAKDN*, *DMC1* for *APOE*, and *LPP*, *ZNF510* for *TREM2*, which reduced the hazard of AD onset more than half. We validated the 27 SR pairs in the external dataset, showing their associations with delayed AD onset and better cognitive outcomes despite neuropathological burden. We further explored the biological mechanism of the SR interactions in AD, implicating lipid and sterol metabolism in glial cells, particularly oligodendrocytes and astrocytes. Our study advances understanding of SR interactions in AD, offering novel therapeutic strategies beyond targeting AD risk loci.

## Supplementary Information

Additional file 1: Figure S1. The workflow of identifying AD risk genes. Figure S2. The distribution of GVB values and their association with AD onset. Figure S3. GVB analysis results in the ADSP cohort. Figure S4. The molecular genetic features of AD risk genes identified by two different methods. Figure S5. Age of onset analysis-significant SR gene pairs. Figure S6. Additive effect of rescuer genes on AD onset in APOE-damaged individuals. Figure S7. Sensitivity analysis of SR pairs across alternative GVB thresholds.

Additional file 2: Table S1. Single-variant analysis results in the ADSP cohort. Table S2. The list of candidate SR gene pairs.

## Data Availability

The dataset from ADSP is available at dbGaP (accession: phs000572.v8.p4; (https://www.ncbi.nlm.nih.gov/projects/gap/cgi-bin/study.cgi?study_id=phs000572.v8.p4) ). The ROSMAP data is available at the AD Knowledge Portal (https://adknowledgeportal.synapse.org/).

## Abbreviations

Aβ: Amyloid beta
AD: Alzheimer’s disease
ADSP: Alzheimer’s disease sequencing project
APP: Amyloid precursor protein
cMAC: Cumulative minor allele count
DLPFC: Dorsolateral prefrontal cortex
FDR: False discovery rate
GVB: Gene-wise variant burden
GWAS: Genome-wide association study
HR: Hazard ratio
MAC: Minor allele count
MAP: Rush memory and aging project
NFT: Neurofibrillary tangle
OPC: Oligodendrocyte precursor cell
pLI: Probability of being loss-of-function intolerant
pLOF: Putative loss-of-function
PPI: Protein-protein interaction
RHS: Rescued-hazard score
RIN: RNA integrity number
ROS: Religious orders study
SIFT: Sorting intolerant from tolerant
SNV: Single nucleotide variant
SR: Synthetic rescue
SRS: Synthetic rescue score
UMI: Unique molecular identifier
WES: Whole exome sequencing
WGS: Whole genome sequencing
