# Supplementary material for: Genome-wide investigation of synthetic rescue interactions in Alzheimer’s disease implicates glial lipid and sterol metabolism
Source: Alzheimers Res Ther. 2026 Apr 11;18:146. doi: 10.1186/s13195-026-02009-4 (PMC13262459; doi:10.1186/s13195-026-02009-4)
Supplement: Supplementary file 1 — Additional file 1: Figure S1. The workflow of identifying AD risk genes. Figure S2. The distribution of GVB values and their association with AD onset. Figure S3. GVB analysis results in the ADSP cohort. Figure S4. The molecular genetic features of AD risk genes identified by two different methods. Figure S5. Age of onset analysis-significant SR gene pairs. Figure S6. Additive effect of rescuer genes on AD onset in APOE-damaged individuals. Figure S7. Sensitivity analysis of SR pairs across alternative GVB thresholds. [file 13195_2026_2009_MOESM1_ESM.docx]

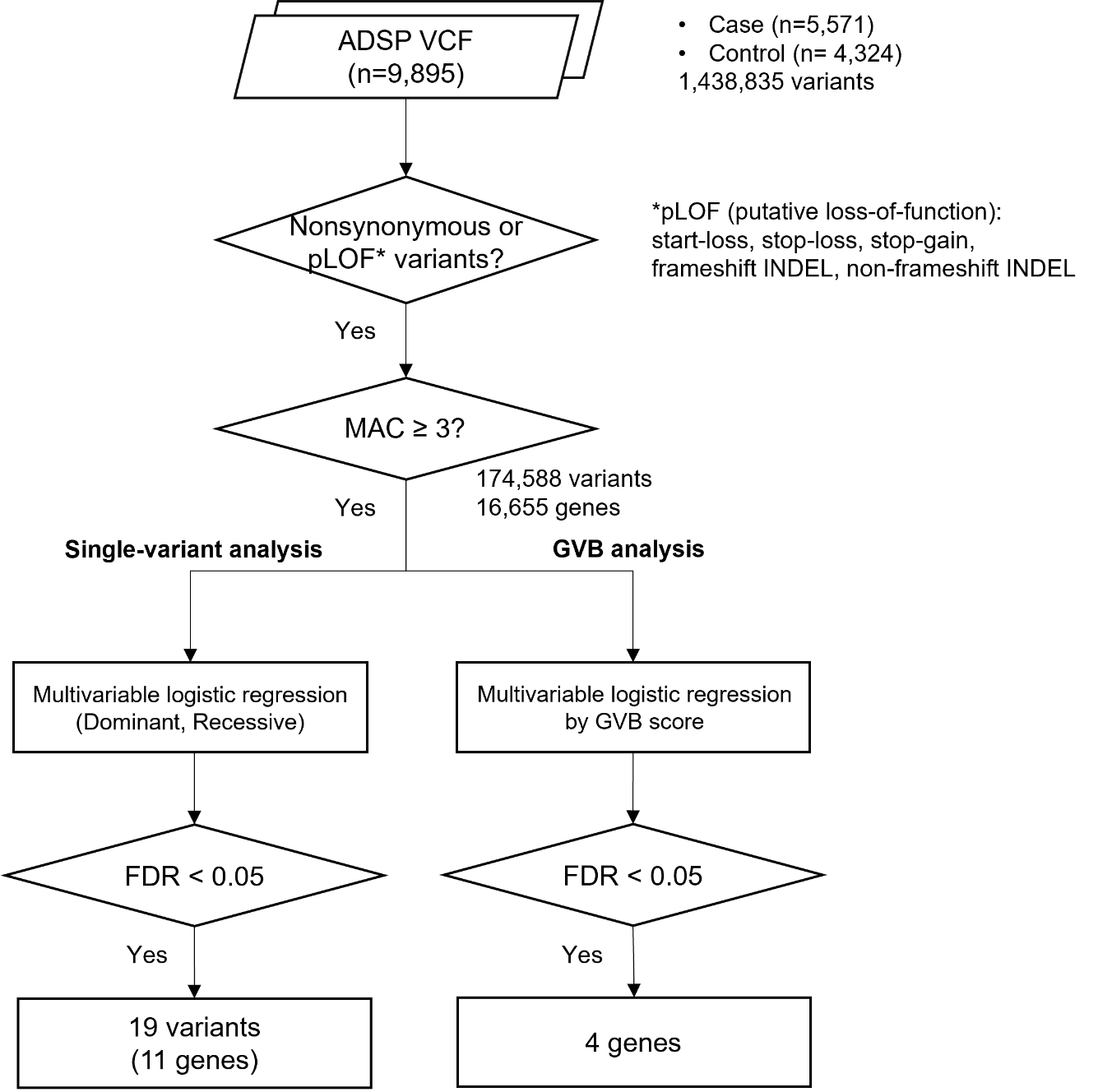


**Fig. S1** **The workflow of identifying AD risk genes**

The flowchart shows the process of identifying AD risk genes in the ADSP cohort. VCF, variant call format; pLOF, putative loss-of-function; MAC, minor allele count; GVB, gene-wise variant burden; FDR, false discovery rate.


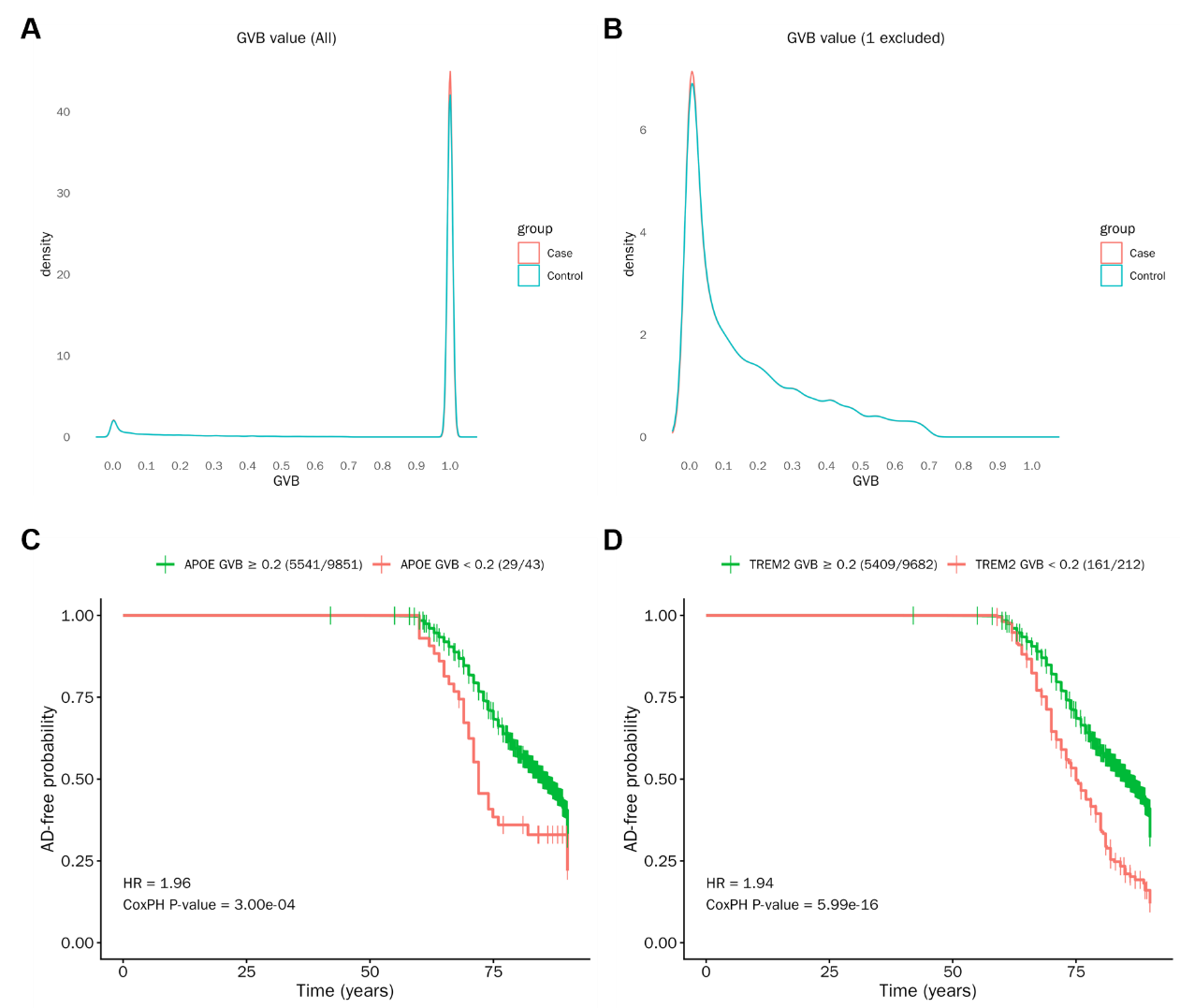


**Fig. S2 The distribution of GVB values and their association with AD onset**

**A-B** The distribution of all GVB values **(A)** and GVB values excluding 1 **(B)** in case and control groups. **C-D** Kaplan-Meier plots comparing AD-free probability between high GVB (GVB ≥ 0.2) and low GVB (GVB < 0.2) groups for well-known AD risk genes, *APOE* **(C)** and *TREM2* **(D)**. Each group is annotated with (The number of AD patients in the group / The total number of individuals in the group). Cox proportional hazard regression adjusting for sex, sequencing center and the top 10 PCs was used to obtain the HR and the p-value.


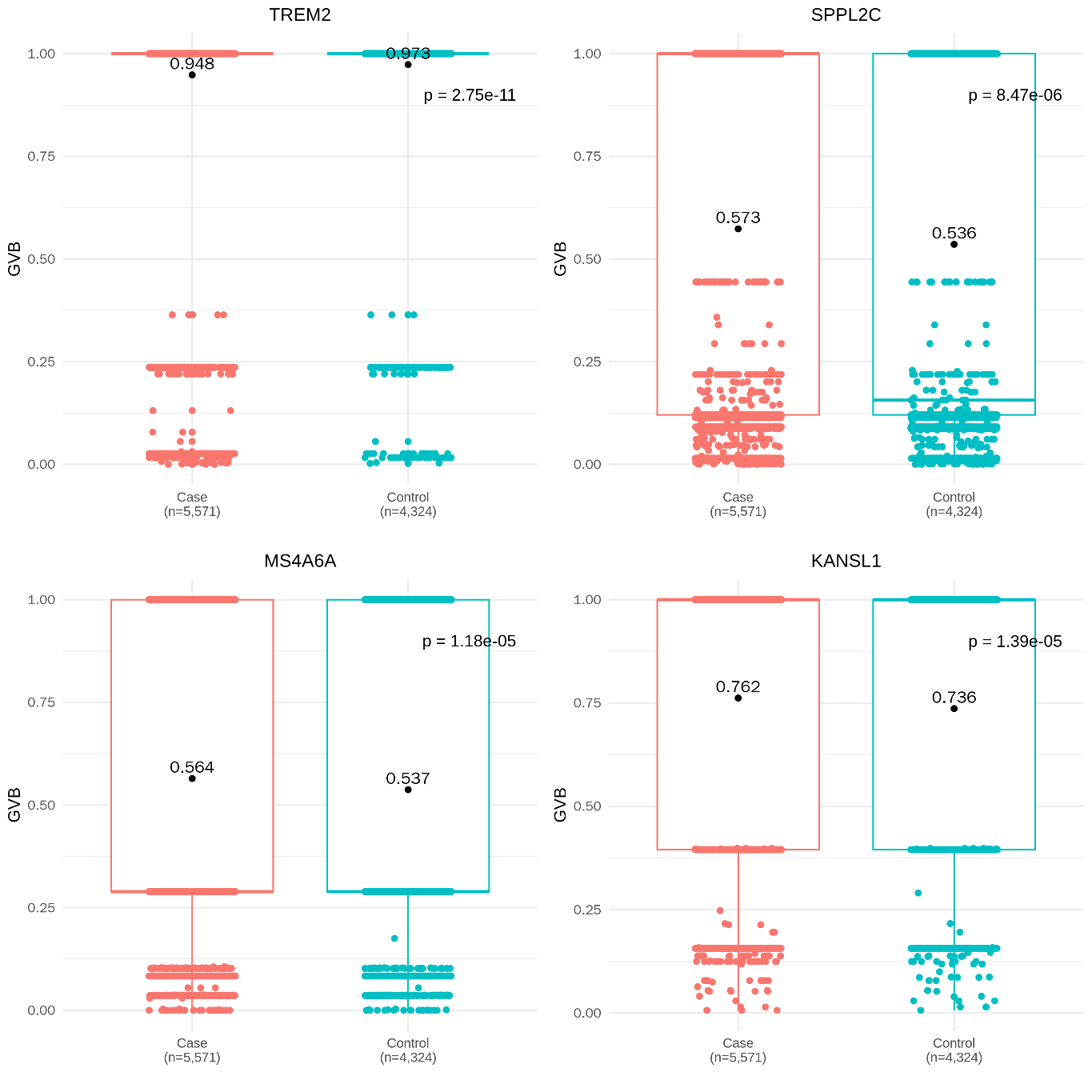


**Fig. S3** **GVB analysis results in the ADSP cohort**

The boxplots show the distribution of GVB values for genes identified by GVB analysis. Black dots represent the mean of GVB in each group. P-values are obtained from multivariable logistic regression.


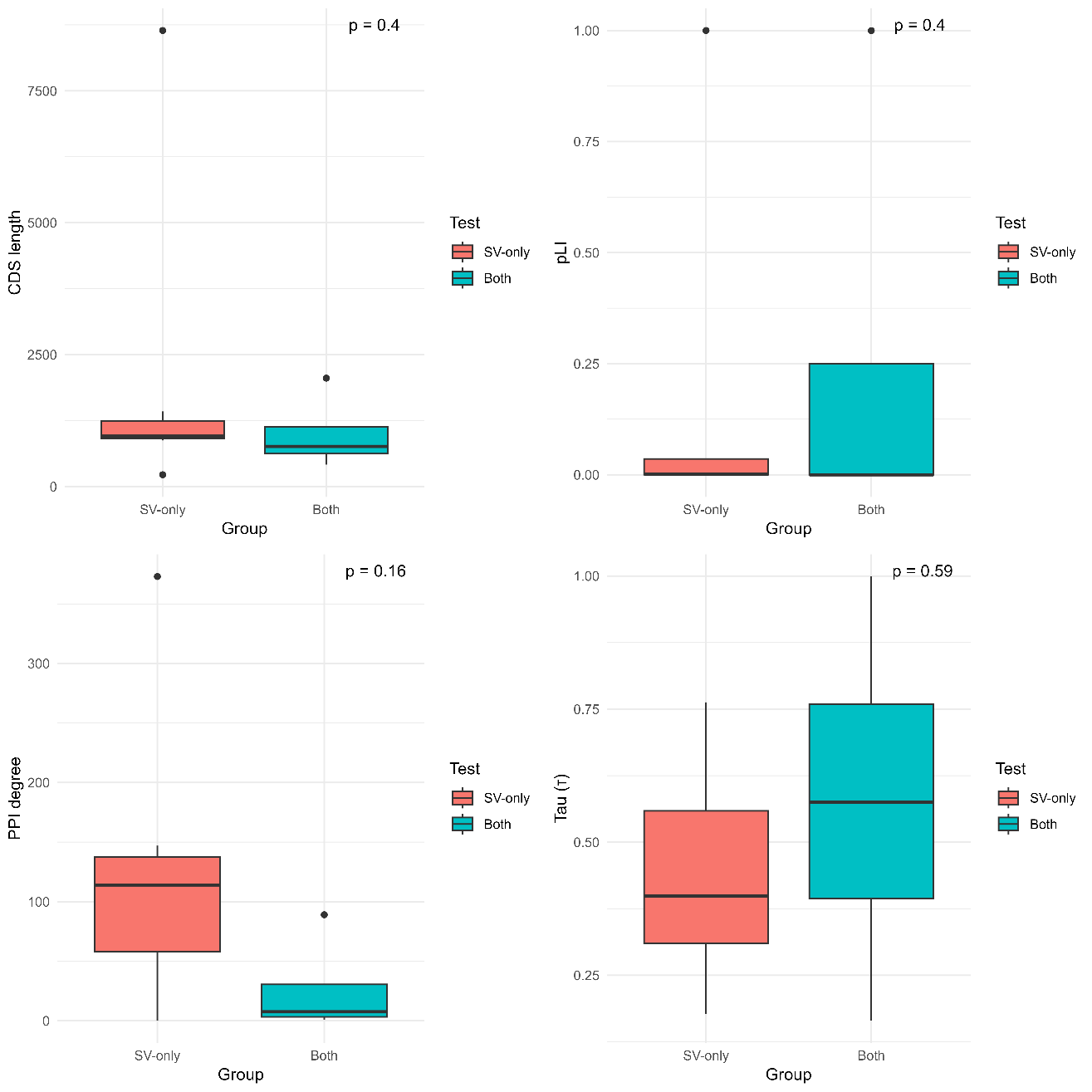


**Fig. S4 The molecular genetic features of AD risk genes identified by two different methods**

Boxplots comparing the molecular genetic features of genes identified exclusively by single-variant analysis (SV-only, red) and genes identified by both single-variant and GVB analyses (Both, blue). Four features indicating gene essentiality are shown: CDS length (top left), pLI score (top right), PPI degree (bottom left), and Tau (bottom right). P-values were calculated using the Wilcoxon rank-sum test.


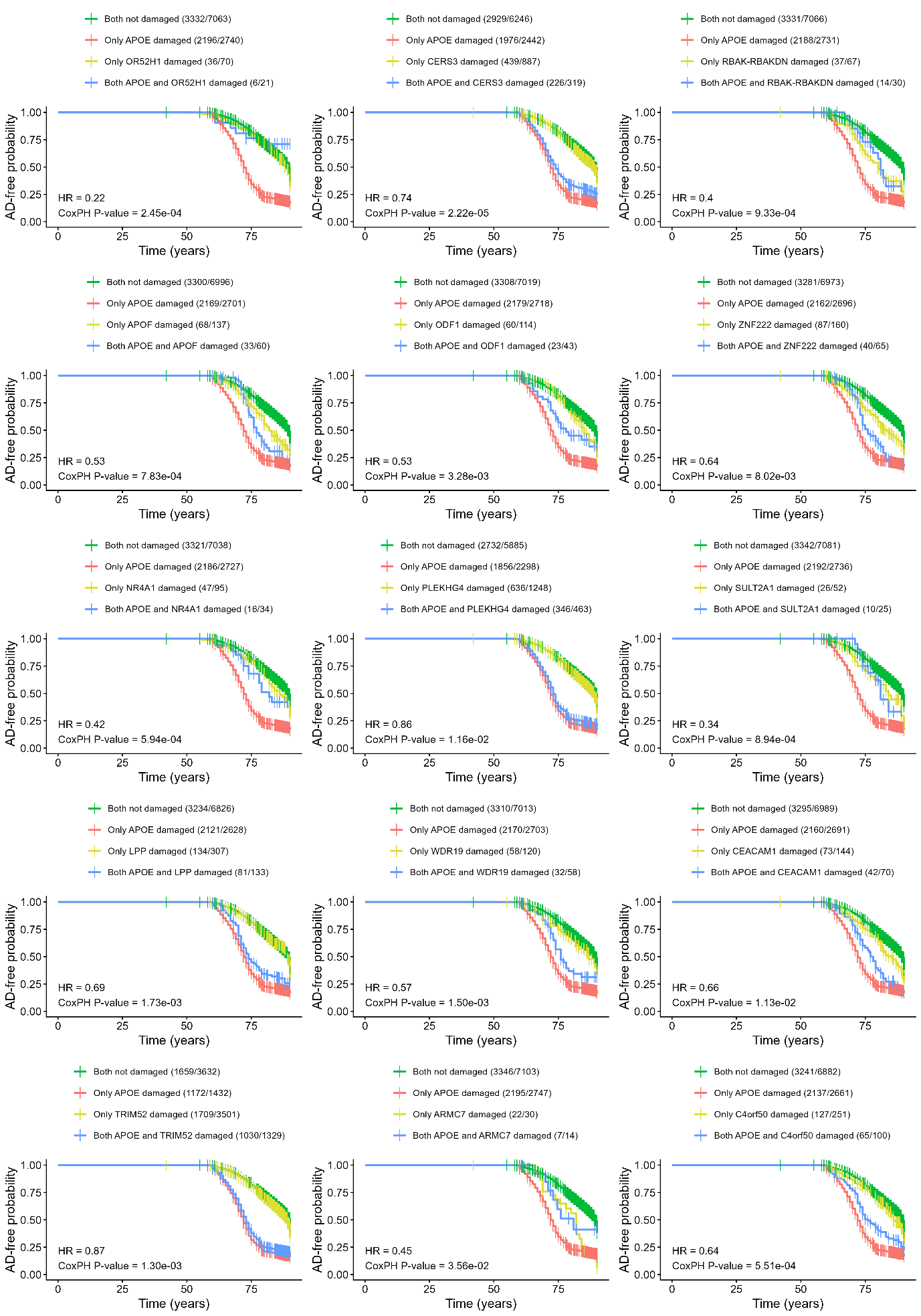


(Continued on next page)
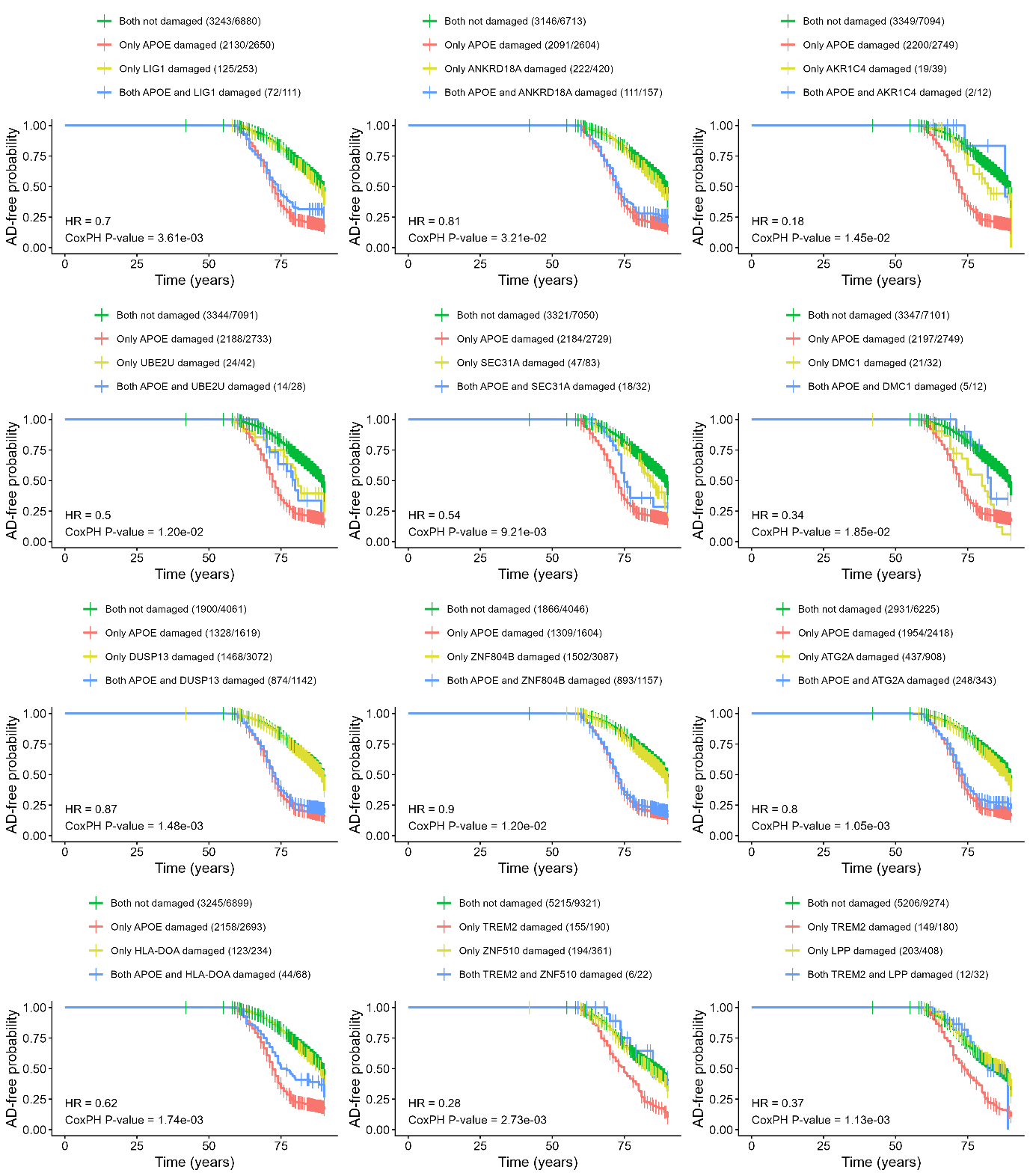


**Fig. S5 Age of onset analysis-significant SR gene pairs**

Kaplan-Meier plots showing 27 SR gene pairs that were significant (HR < 1, FDR < 0.05) in age of onset analysis. Cox proportional hazards regression was used to obtain the HRs and the p-values. Each group is annotated with (The number of AD patients in the group / The total number of individuals in the group).


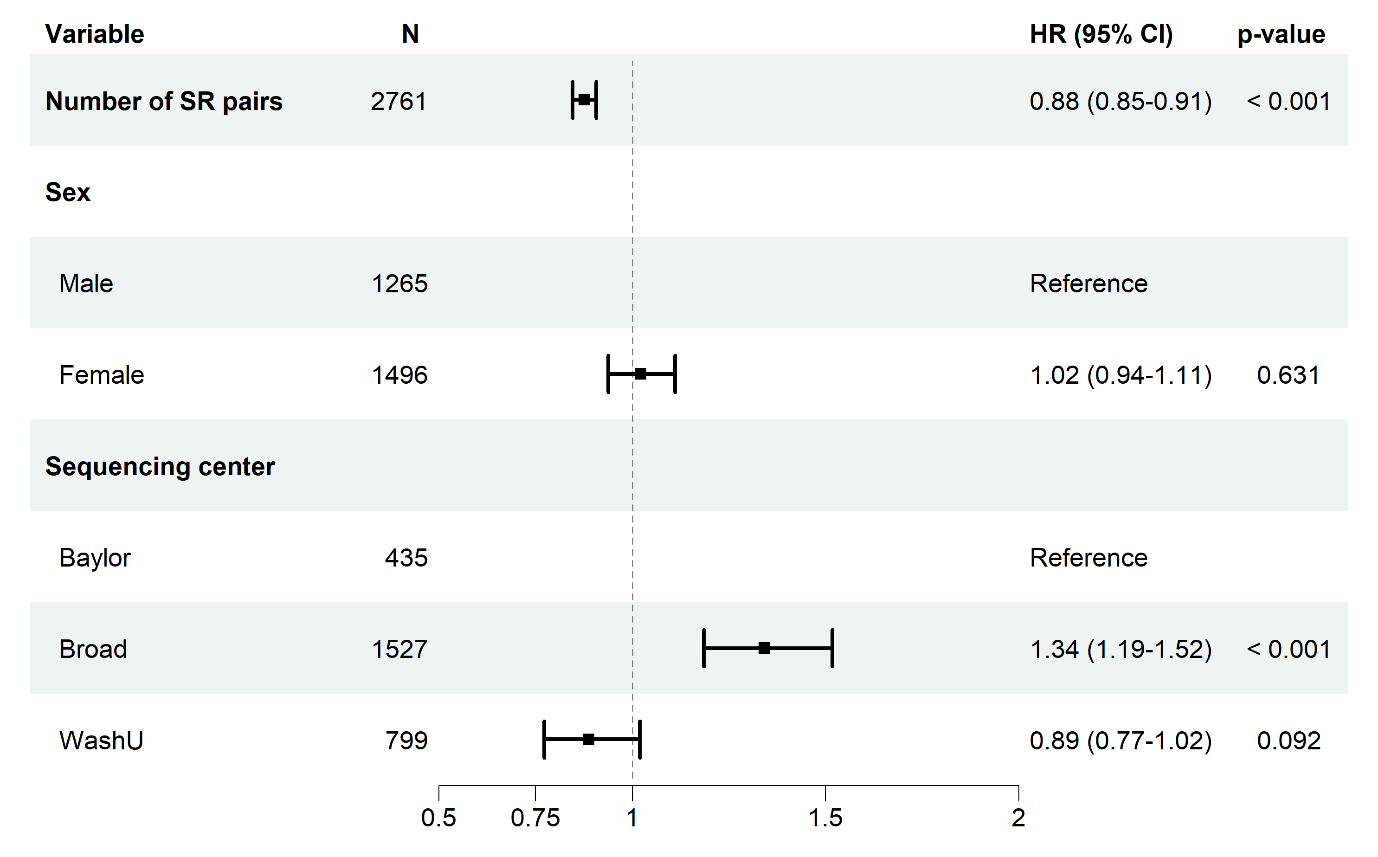


**Fig. S6 Additive effect of rescuer genes on AD onset in *APOE*-damaged individuals**

Forest plot from Cox proportional hazards regression showing the effect of the number of SR pairs for *APOE* on AD onset. Only 2,761 rs429358 carriers (classified as *APOE*-damaged) were included.


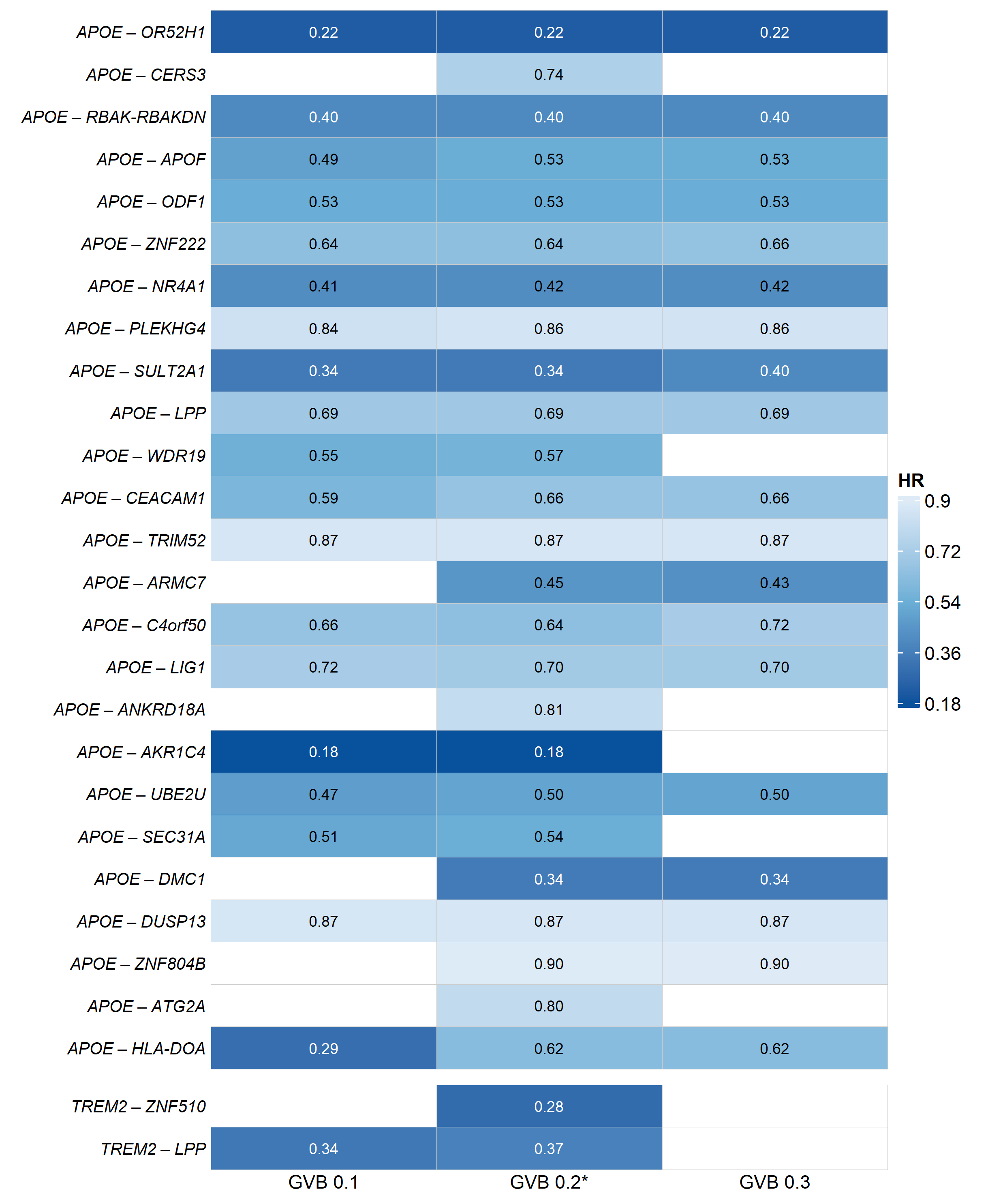


**Fig. S7 Sensitivity analysis of SR pairs across alternative GVB thresholds**

SR pairs identified at GVB threshold of 0.2 were evaluated for their replication at alternative thresholds of 0.1 and 0.3. Only significant HRs obtained from age of onset analysis are shown. *Primary GVB threshold.
